# *MicroRNA-130b* functions as an oncomiRNA in non-small cell lung cancer by targeting tissue inhibitor of metalloproteinase-2

**DOI:** 10.1038/s41598-019-43355-8

**Published:** 2019-05-06

**Authors:** Takayuki Hirono, Kentaro Jingushi, Toshiyuki Nagata, Masami Sato, Kentaro Minami, Masaya Aoki, Aya Harada Takeda, Tadashi Umehara, Hiroshi Egawa, Yoshino Nakatsuji, Kaori Kitae, Yuko Ueda, Hiroaki Hase, Masatatsu Yamamoto, Yoshinari Shinsato, Kohichi Kawahara, Tatsuhiko Furukawa, Kazutake Tsujikawa

**Affiliations:** 1 Laboratory of Molecular and Cellular Physiology, Graduate School of Pharmaceutical Sciences, Osaka University, 1-6 Yamadaoka, Suita, Osaka 565-0871 Japan; 2 Department of General Thoracic Surgery, Graduate School of Medical and Dental Sciences, Kagoshima University, 8-35-1 Sakuragaoka, Kagoshima, Kagoshima 890-8520 Japan; 3 Department of Molecular Oncology, Graduate School of Medical and Dental Sciences, Kagoshima University, 8-35-1 Sakuragaoka, Kagoshima, Kagoshima 890-8544 Japan; 4 Center for the Research of Advanced Diagnosis and Therapy of Cancer, Graduate School of Medical and Dental Sciences, Kagoshima University, Kagoshima, 890-8544 Japan

**Keywords:** Oncogenes, Non-small-cell lung cancer

## Abstract

Non-small cell lung cancer (NSCLC) is the most frequent cause of cancer-related death worldwide. Although many molecular-targeted drugs for NSCLC have been developed in recent years, the 5-year survival rate of patients with NSCLC remains low. Therefore, an improved understanding of the molecular mechanisms underlying the biology of NSCLC is essential for developing novel therapeutic strategies for the treatment of NSCLC. In this study, we examined the role of *miR-130b* in NSCLC. Our results showed that high expression of *miR-130b* in clinical specimens was significantly associated with poor overall survival in patients with NSCLC. Moreover, *miR-130b* expression was significantly increased in NSCLC clinical specimens from patients with vascular and lymphatic invasion. Consistent with this, overexpression of *miR-130b* promoted invasion and matrix metalloproteinase-2 (MMP-2) activity in A549 cells. Argonaute2 immunoprecipitation and gene array analysis identified tissue inhibitor of metalloproteinase-2 (TIMP-2) as a target of *miR-130b*. Invasion activity promoted by *miR-130b* was attenuated by TIMP-2 overexpression in A549 cells. Furthermore, TIMP-2 concentrations in serum were inversely correlated with relative *miR-130b* expression in tumor tissues from the same patients with NSCLC. Overall, *miR-130b* was found to act as an oncomiR, promoting metastasis by downregulating TIMP-2 and invasion activities in NSCLC cells.

## Introduction

Lung cancer is the most common cause of cancer-related death worldwide, causing an estimated 1.6 million deaths each year. Approximately 85% of patients with lung cancer have the histological subtype known as non-small cell lung cancer (NSCLC), in which lung adenocarcinoma and lung squamous cell carcinoma are the most common subtypes^1,2^. Although several therapeutic agents for NSCLC have been developed, the prognosis of patients with NSCLC is still poor, and the 5-year survival rate is less than 18%. This low survival rate may be explained by the observation that most cases are diagnosed as a late stage, when lung cancer cells have already metastasised to distant organs^3–5^.

To break away from the primary tumor and initiate the metastatic process, cancer cells must acquire the ability to migrate and invade^6,7^. Matrix metalloproteinases (MMPs) are proteases involved in extracellular matrix remodelling^8^. In particular, MMP-2 plays a key role in tumor invasion and metastasis by degrading type IV collagen, which is a major component of the basal membrane^9^. High expression of MMP-2 has been reported in several cancers and is a poor prognostic factor in patients with NSCLC^10–14^. Pro-MMP-2, an inactivated form of MMP-1, is activated on the cell surface by membrane-tethered membrane type 1-MMP (MT1-MMP), in a process regulated by tissue inhibitor of metalloproteinase (TIMP)-2. A complex of active membrane-tethered MT1-MMP and TIMP-2 binds to pro-MMP-2, enabling pro-MMP-2 to be activated by MT1-MMP^15^.

MicroRNAs (miRNAs) are small noncoding RNA molecules 20–25 nucleotides in length. These molecules regulate gene expression through translational repression or degradation of mRNA by binding to the 3′-untranslated region (3′-UTR) of target mRNAs^16^. Each miRNA typically targets approximately 200 genes^17,18^. Because 30–60% of human genes can be regulated by miRNAs^19,20^, these molecules have the potential to modulate various cellular processes, such as cell growth, migration, invasion, apoptosis, and angiogenesis^21^. Our previous study showed that the *miR-130* family, including *miR-130b*, *miR-301a*, and *miR-301b*, is highly expressed in bladder cancer specimens and functions as an oncogenic miRNA family by promoting the migration and invasion of bladder cancer cells^22^. Moreover, *miR-130* family-targeted LNA oligonucleotides were found to suppress tumor growth in an *in vivo xenograft* model^23^.

In this study, we evaluated the expression and roles of *miR-130b* in NSCLC. Our results provided important insights into the molecular pathogenesis of NSCLC and suggested that *miR-130b* may function as an oncogenic miRNA in NSCLC.

## Results

### High *miR-130b* expression was correlated with poor overall survival in patients with NSCLC

**Figure 1:**
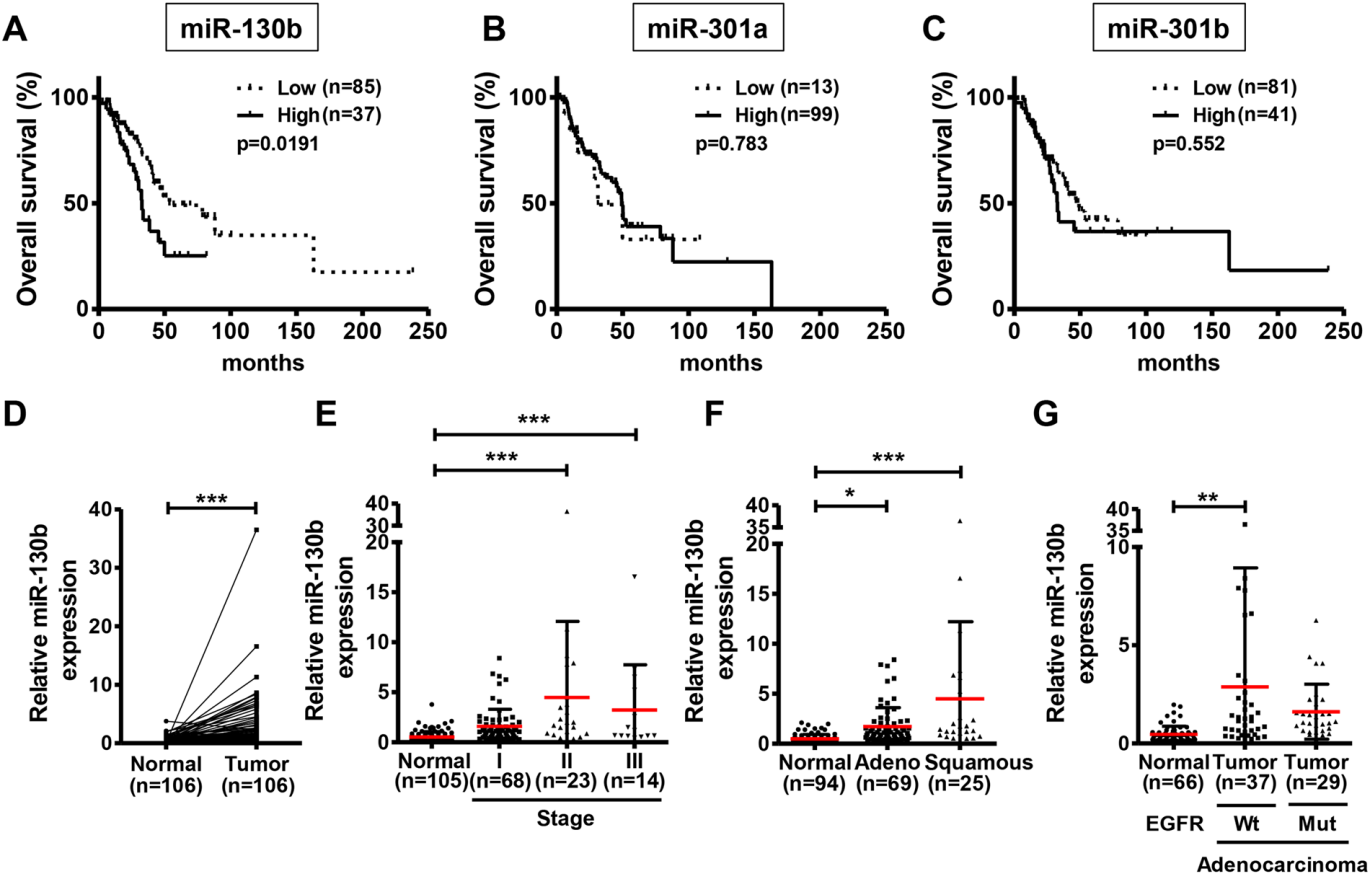
High *miR-130b* expression was correlated with poor overall survival in NSCLC clinical specimens. (**A**–**C**) TCGA database analysis of *miR-130b*, *miR-301a*, and *miR-301b* in patients with adenocarcinoma. Overall survival analysis in patients with high (copy number: 1) and low (copy number: −1) *miR-130* family expression was analysed by Kaplan-Meier analysis with log-rank tests. The number of patients analysed is indicated in parentheses. *miR-130b* expression levels were measured by real-time qPCR and were compared among normal and tumor tissues (**D**) and tumor stages (**E**) in NSCLC clinical specimens, tumor subtypes in specimens with adenocarcinoma or squamous cell carcinoma (**F**), status of epidermal growth factor receptor gene mutation in specimens with adenocarcinoma (**G**). Relative *miR-130b* expression normalized to U6 snRNA is shown. Data are means ± standard deviations. **p < 0.01 and ***p < 0.001 for t-tests (**D**) and one-way analysis of variance (post-hoc Bonferroni multiple comparison tests) (**E**–**G**).

Using The Cancer Genome Atlas (TCGA) database, we first investigated the relationship between expression of the *miR-130* family and prognosis of patients with NSCLC. Although there was no significant relationship between *miR-130* family expression and the prognosis of patients with squamous cell carcinoma (Supplementary Fig. 1A–C), adenocarcinoma patients with high *miR-130b* expression had significantly poorer overall survival than those with low *miR-130b* expression (Fig. 1A). In contrast, there were no significant relationships between the expression of *miR-301a* or *miR-301b* and overall survival in patients with adenocarcinoma (Fig. 1B,C). Therefore, we focused on *miR-130b* in subsequent analyses. To confirm the expression of *miR-130b* in NSCLC clinical specimens, we performed real-time quantitative polymerase chain reaction (qPCR) analysis using matched pair samples of NSCLC tissues and normal adjacent lung tissues. We found that *miR-130b* expression was significantly higher in NSCLC tissues than in normal adjacent lung tissues (Fold-change 5.0, p < 0.001, Fig. 1D). *miR-130b* expression in NSCLC tissues tended to increase as the cancer stage increased (Fig. 1E). Interestingly, *miR-130b* expression was high in NSCLC tissues, regardless of histologic subtypes (Fig. 1F) and of the presence or absence of epidermal growth factor receptor gene mutation (Fig. 1G, Table 1) in adenocarcinoma specimens. These results suggested that *miR-130b* had an important role in NSCLC.

**Table 1:** NSCLC clinical samples used in Figs 1D–G, 2D–F.

Age (y)		Gender	
median	71	male	65
range	50–87	female	41
**Histological subtype**		**Clinical stage**	
adenocarcinoma	69	I	68
squamous	25	II	23
cell carcinoma		III	14
others	12	unknown	1
**EGFR mutation (adenocarcinoma)**		**Vessel cancer cell (v) invasion**	
exon 18	1	v (−)	70
exon 19	13	v (+)	36
exon 21	15		
wild-type	37	**Pleura cancer cell (pl) invasion**	
unknown	3	pl (−)	70
**Lymphatic cancer cell (ly) invasion**		pl (+)	35
ly (−)	60	unknown	1
ly (+)	45		
unknown	1		

### *miR*-*130b* promoted invasion activity in NSCLC cells

**Figure 2:**
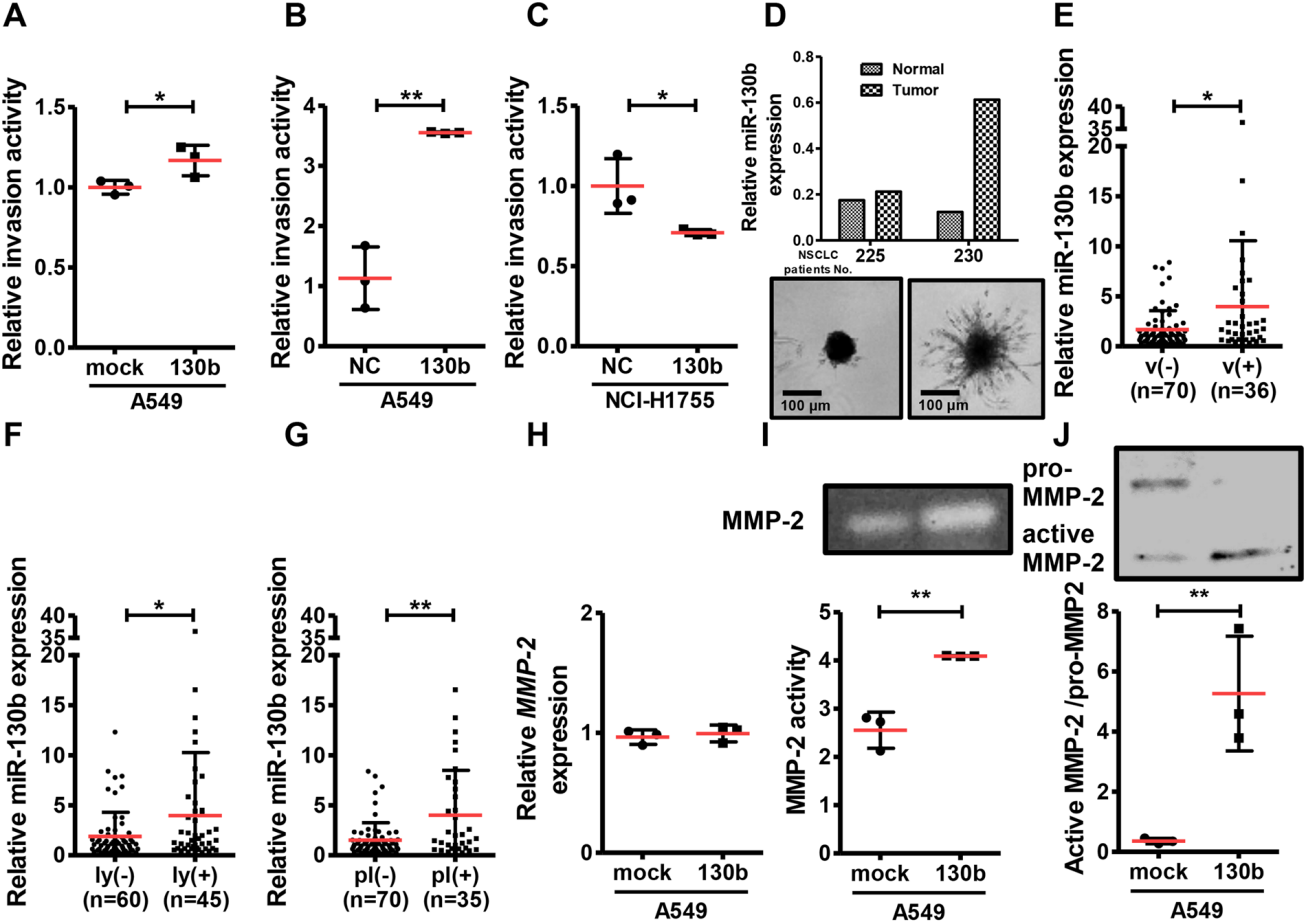
*miR-130b* overexpression promoted invasion and MMP-2 activities in A549 cells. A549 cells stably overexpressing *miR-130b* or mock control (mock) (**A**), A549 cells transfected with the *miR-130b* mimic (130b) or negative control miRNA mimic (NC) (**B**), and NCI-H1755 cells transfected with the *miR-130b* inhibitor (130b) or negative control miRNA inhibitor (NC) (**C**) were subjected to invasion assays. The transfected cells were added to the upper chambers of Matrigel-coated transwell membrane inserts, and the lower chambers were filled with medium. Cells were cultured for 20 h (A549 cells), or 48 h (NCI-H1755). Fluorescence derived from invasive cells was measured. Data are means ± standard deviations of three independent experiments. *p < 0.05 and **p < 0.01 for t-tests. (**D**) *miR-130b* expression level was examined by real-time qPCR. Primary cultured NSCLC cells were seeded into Nunclon Sphera 96U-well plates and incubated for 48 h. The medium was replaced with that containing Matrigel and incubated for 72 h. The expression of *miR-130b* was compared in NSCLC specimens with or without vascular invasion (**E**), lymphatic invasion (**F**), and pleura cancer cell invasion (**G**). Relative expression normalized to U6 snRNA is shown (means ± standard deviations). *p < 0.05 for t-tests. (**H**) *MMP-2* levels in A549 cells stably overexpressing *miR-130b* or control vector (mock) were analysed by real-time qPCR. The relative expression of *MMP-2* normalized to *GAPDH* is shown (means ± standard deviations) from three independent experiments. Conditioned medium from A549 cells stably overexpressing *miR-130b* or control vector (mock) were subjected to gelatine-zymography (**I**) and western blotting analysis with anti-MMP-2 antibodies (**J**). Uncropped zymography and Western blot data is shown in Supplementary Fig. S9. Representative results from three independent experiments are shown. Data are means ± standard deviations from three independent experiments. *p < 0.05 and **p < 0.01 for t-tests.

To investigate the biological functions of *miR-130b*, we first determined the expression level of *miR-130b* in NSCLC cell lines. Among 20 adenocarcinoma and squamous cell carcinoma cell lines, the lowest expression of *miR-130b* was found in A549 cells (Supplementary Fig. 2A). We then established A549 cells stably overexpressing *miR-130b* (Supplementary Fig. 2B) and examined the effects of *miR-130b* on tumor-promoting phenotypes in the cells. Although *miR-130b* overexpression had no significant effect on cell growth or migration (Supplementary Fig. 3A–C), invasion activity was upregulated in A549 cells stably overexpressing miR-130b (Fig. 2A). Next, we transfected the miR-130b mimic into A549 (not stably overexpressing miR-130b), NCI-H520 and NCI-H1975 cells, which show the lowest endogenous *miR-130* expression, or *miR-130b* inhibitor into NCI-H1755 cells, which exhibit the highest endogenous *miR-130* expression, of all NSCLC cell lines examined in this study (Supplementary Fig. 4A–D). Transfection with the *miR-130b* mimic markedly upregulated the invasion activity of A549 cells (Fig. 2B) and NCI-H520 cells (Supplementary Fig. 5), suggesting that *miR-130b* promotes cell invasion activity not only in adenocarcinoma cells but also in squamous cell carcinoma cells. In contrast, transfection with the *miR-130b* inhibitor downregulated the invasion activity of NCI-H1755 cells (Fig. 2C), without affecting cell proliferation (Supplementary Fig. 6). Importantly, primary cultured NSCLC cells with high *miR-130b* expression (No. 230) showed a more obvious invasive phenotype than cells with low *miR-130b* expression (No. 225) in the 3D invasion assay (Fig. 2D). Importantly, tumor samples from patients with vascular invasion, lymphatic invasion and pleura cancer cell invasion had higher *miR-130b* expression than those without invasion (Fig. 2E–G). Because high expression of MMP-2, which functions as a key enzyme in tumor cell invasion, has been reported as a prognostic factor in NSCLC^12^, we next examined the effects of *miR-130b* on MMP-2 activity in *miR-130b*-overexpressing A549 cells. Although *MMP-2* mRNA levels were not altered (Fig. 2H), *miR-130b*-overexpressing A549 cells showed increased MMP2 activity in gelatine-zymography assays (Fig. 2I) and increased MMP-2 expression in western blot analysis using anti-MMP-2 antibodies (Fig. 2J) compared with that in mock-transfected A549 cells. The extracellular protein level was nearly identical between mock cells and *miR-130b*-overexpressing A549 cells (Supplementary Table 1). These results suggested that *miR-130b* promoted invasion in NSCLC cells via upregulation of MMP-2 activity.

### *miR-130b* promoted invasion via targeting *TIMP-2* in NSCLC cells

To identify target genes of *miR-130b*, we conducted an argonaute2 (Ago2) immunoprecipitation (IP) assay using A549 cells transfected with an empty vector or *miR-130b* expression vector (Supplementary Fig. 7A). mRNAs immunoprecipitated with anti-Ago2 antibodies were then subjected to gene array analysis. Candidate target genes of *miR-130b* were extracted by miRNA target prediction algorithms (miRbase, TargetScan, and miRWalk; Supplementary Table 2). Among the identified genes, we focused on *TIMP-2*, which functions as an inhibitor of MMP-2 activity, as a candidate *miR-130b* target gene in NSCLC cells.

**Figure 3:**
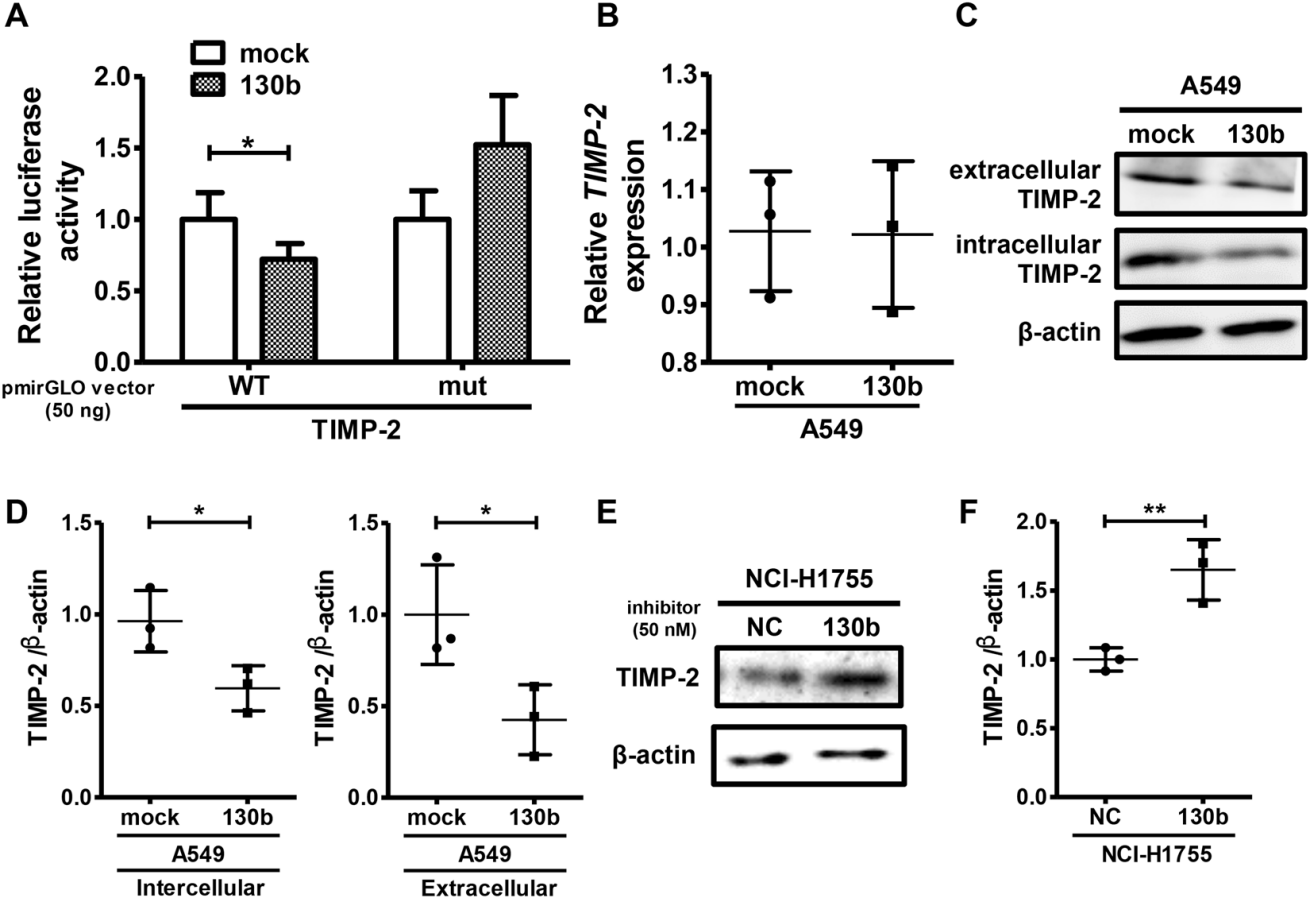
*miR-130b* targeted TIMP-2 in NSCLC cells. (**A**) A549 cells stably overexpressing *miR-130b* were transfected with a luciferase reporter construct containing the predicted *miR-130b*-binding site (WT) or the mutated predicted *miR-130b*-binding site (mut) in the *TIMP-2* 3′-UTR. Data are means ± standard deviations of more than five independent experiments. *p < 0.05 for one-way analysis of variance followed by post-hoc Bonferroni multiple comparison tests. (**B**) A549 cells stably overexpressing *miR-130b* (130b) or control vector (mock) were subjected to real-time qPCR analysis of *TIMP-2*. Relative expression of *TIMP-2* normalized to *GAPDH* is shown (means ± standard deviations) from three independent experiments. (**C**,**D**) A549 cells stably overexpressing *miR-130b* (130b) or control vector (mock) were subjected to western blotting with anti-TIMP-2 antibodies. Representative results from three independent experiments are shown. Representative results of three independent experiments are shown for (**C**). The numbers indicate the relative expression of TIMP-2 compared to that in NC, as analyzed by densitometry. *p < 0.05 for t-tests. Uncropped western blot data is shown in Supplementary Fig. 10. (**E**,**F**) NCI-H1755 cells were transfected with the negative control inhibitor (NC) or *miR-130b* inhibitor (130b) for 48 h, and whole-cell lysates were subjected to western blotting with anti-TIMP-2 antibodies. Representative results of three independent experiments are shown. The numbers indicate the relative expression of TIMP-2 compared to that in NC, as analysed by densitometry. *p < 0.05 for t-tests. Uncropped western blot data is shown in Supplementary Fig. 10.

To confirm whether *TIMP-2* is a target gene of *miR-130b*, we transfected cells with a luciferase reporter vector harbouring a sequence containing the predicted *miR-130b*-binding site in the 3′-UTR of human *TIMP-2* (wild-type TIMP-2) or that containing the mutated *miR-130b*-binding site (mutant TIMP-2) in A549 cells (Supplementary Fig. 7B). As shown in Fig. 3A, transfection with the wild-type TIMP-2 vector significantly decreased luciferase activity in *miR-130b*-overexpressing A549 cells compared with that in mock control A549 cells. In contrast, *miR-130b* overexpression had no significant effect on luciferase activity in mutant TIMP-2-transfected A549 cells (Fig. 3A). Although *miR-130b* overexpression had no significant effect on *TIMP-2* mRNA levels (Fig. 3B), western blot analysis showed decreased protein levels of both extracellular and intracellular TIMP-2 in *miR-130b*-overexpressing A549 cells (Fig. 3C,D). Moreover, transfection with the *miR-130b* mimic decreased TIMP-2 protein levels in A549, NCI-H520 and NCI-H1975 cells (Supplementary Fig. 8A–D), while *miR-130b* mimic or inhibitor had no effect on *TIMP-2* mRNA expression in the cells (Supplementary Fig. 9A–D). Conversely, transfection with a *miR-130b* inhibitor upregulated TIMP-2 protein expression in NCI-H1755 cells (Fig. 3E,F), indicating that *TIMP-2* was a target of *miR-130b* in NSCLC cells.

**Figure 4:**
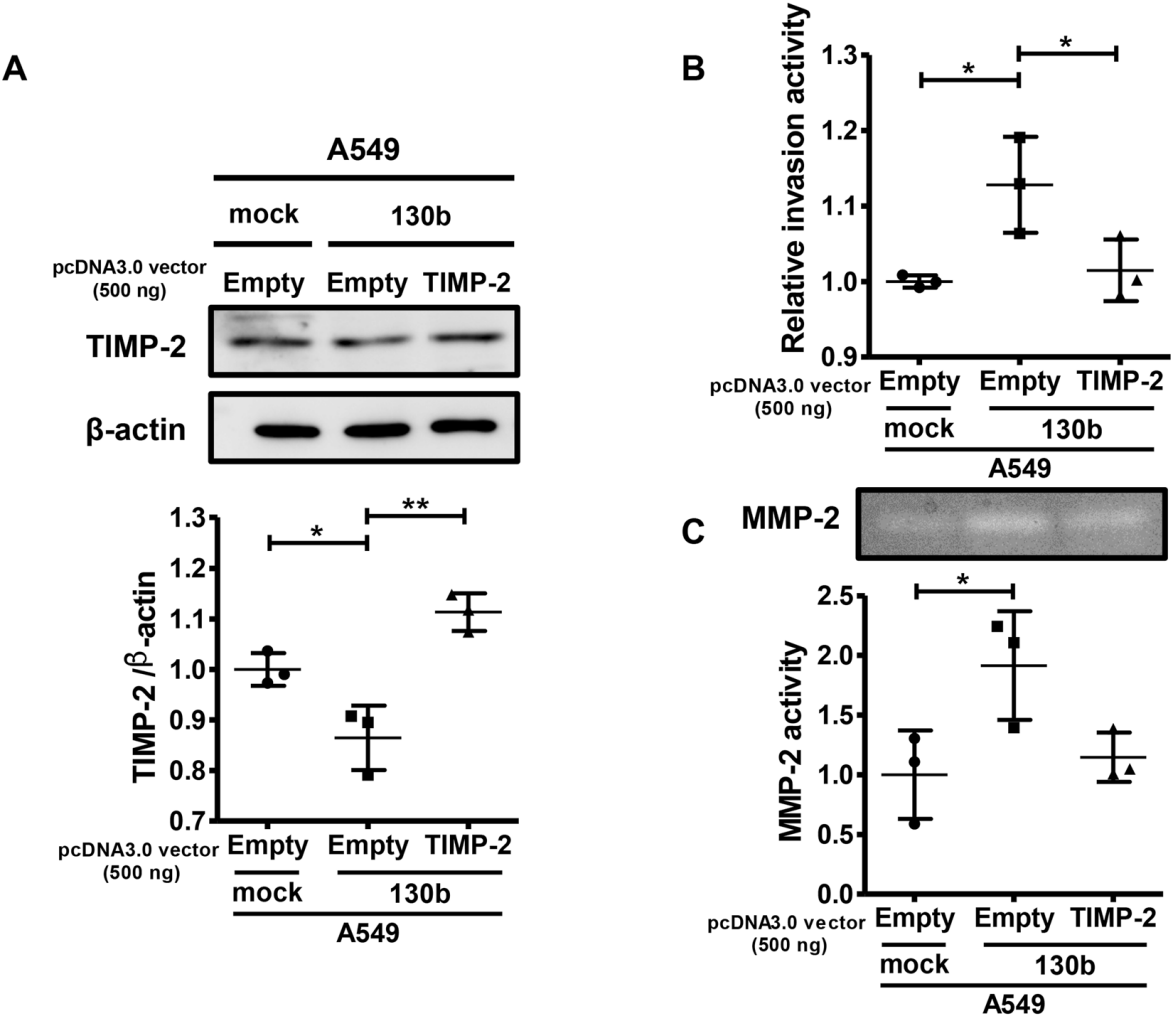
*miR-130b* promoted invasion by targeting TIMP-2 in A549 cells. Control (mock) or *miR-130b*-overexpressing A549 cells (130b) were transfected with the control vector (empty) or TIMP-2 expression vector (TIMP-2) for 48 h. Cells were then subjected to western blot analysis (**A**), invasion assays (**B**), and gelatine-zymography (**C**). Uncropped Western blot and zymography data are shown in Supplementary Fig. 10. Representative results from three independent experiments are shown. The numbers indicate the relative expression of TIMP-2 compared to that of control (mock/empty) A549 cells, as analyzed by densitometry (**A**,**C**). Data are the means ± standard deviations of three independent experiments in A, B, and C. *p < 0.05 for one-way analysis of variance followed by post-hoc Bonferroni multiple comparison tests.

Next, we examined whether promotion of invasion activity by *miR-130b* overexpression was related to a decrease in TIMP-2 expression. The effects of *miR-130b* on invasion activity were attenuated by TIMP-2 overexpression in A549 cells (Fig. 4A,B). Moreover, TIMP-2 overexpression blocked the effects of *miR-130b* on MMP-2 activity in A549 cells (Fig. 4C), suggesting that *miR-130b* promoted cell invasion activity by downregulating TIMP-2 protein expression and upregulating MMP-2 activity in NSCLC cells.

### Serum *TIMP-2* concentrations were inversely correlated with tumor *miR-130b* expression levels in patients with NSCLC

**Figure 5:**
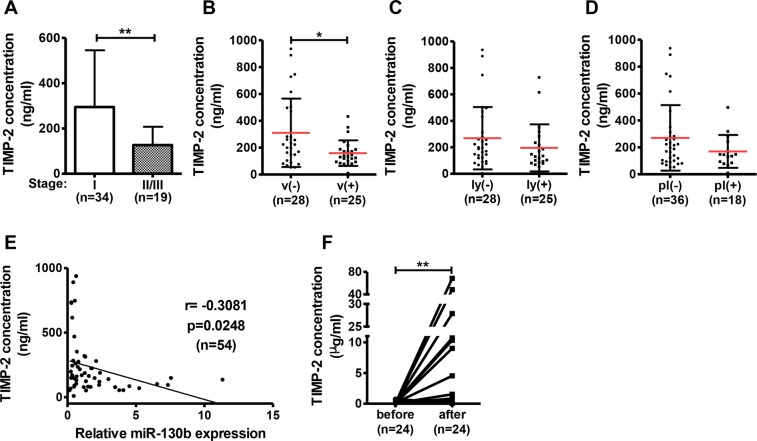
TIMP-2 concentrations in serum were inversely correlated with *miR-130b* expression levels in tumor tissues from patients with NSCLC. Serum concentrations of TIMP-2 were compared according to tumor stage (**A**), with or without vascular (**B**), lymphatic (**C**), and pleura cancer cell invasion (**D**). (**E**) Correlations between TIMP-2 concentrations in serum and relative *miR-130b* expression in tumor tissues of patients with NSCLC were evaluated. (**F**) TIMP-2 concentration in serum was compared before or after operation. Data are means ± standard deviations. *p < 0.05 and **p < 0.01 for t-tests.

Finally, we examined the relationship between TIMP-2 concentrations in the serum and *miR-130b* expression in tumor tissues from patients with NSCLC. Enzyme-linked immunosorbent assays (ELISAs) showed that TIMP-2 concentrations in the serum of patients with NSCLC were significantly lower in patients with stage II/III cancer than in patients with stage I cancer (Fig. 5A). Although no significant relationship was observed between the presence or absence of lymphatic invasion or pleura cancer cell invasion (Fig. 5C,D), TIMP-2 concentrations were significantly lower in serum from patients with NSCLC with vascular invasion of tumor cells than in those patients without invasion (Fig. 5B). Importantly, an inverse correlation was observed between TIMP-2 concentrations in serum and *miR-130b* expression levels in tumor tissues of the same patients with NSCLC (r = −0.3081, *p* = 0.0248; Fig. 5E). Moreover, the TIMP-2 concentrations were significantly higher in the sera of patients after operation than before operation (Fig. 5F).

## Discussion

In the present study, based on the significant correlation between high *miR-130b* expression and poor overall survival in patients with NSCLC, we demonstrated that *miR-130b* increased invasion activity by directly targeting TIMP-2 not only in adenocarcinoma cells but also in squamous cell carcinoma cells using loss-of function and gain-of function experiments. Moreover, we showed that *miR-130b* expression levels in tumor tissues and TIMP-2 concentrations in serum from the same patients were inversely correlated.

In this study, we found that *TIMP-2* was a target of *miR-130b* using Ago2-IP assays, gene array analysis, and target prediction algorithms. TIMP-2 is a member of the TIMP family and functions as an inhibitor of MMP^24^. TIMP-2 characteristically inhibits MMP-2 activation by forming a complex with pro-MMP-2. Under normal physiological conditions, the expression of TIMP-2 is strictly controlled and prevents excessive extracellular matrix reorganisation by MMP-2^25^. Conversely, in cancer tissues, including NSCLC, the expression of TIMP-2 is constantly low, thereby promoting the invasion of cancer cells^26,27^. Our data also indicated that the high expression of *miR-130b* activated MMP-2 by downregulating TIMP-2, leading to invasion of NSCLC cells *in vitro*.

Vascular invasion of lung cancer cells is often observed in low-stage tumors in patients with NSCLC^28^. Although metastasis via the lymphatic route usually takes a longer time for establishment of distant metastasis, tumor cell spreading via blood vessels occurs early during the metastatic process^29^. Our data showed that high expression of *miR-130b* was significantly upregulated in tumor tissues with vascular cancer cell invasion in patients with NSCLC. Moreover, a 3D gel invasion assay using primary cultured NSCLC cells revealed upregulated invasion activity when miR-130b expression was high (Fig. 2D). Therefore, high *miR-130b* expression may function to promote metastasis via targeting TIMP-2 expression and subsequent upregulation of the active form of MMP-2 during the early stages of NSCLC.

Increased TIMP-2 levels in tumor tissues are correlated with a favourable prognosis in patients with NSCLC^30^. TIMP-2 has various functions in addition to MMP-2 inhibition^31^. For example, TIMP-2 inhibits proliferation, migration, and angiogenesis of endothelial cells^32–35^. Moreover, TIMP-2 suppresses vascular permeability via the integrin α3β1/Shp-1/cAMP/protein kinase A pathway independently of MMP inhibition in human vascular endothelial cells^36^. Therefore, elevated *miR-130b* expression may increase the permeability of surrounding vascular endothelial cells via downregulation of TIMP-2 levels in the tumor microenvironment, leading to subsequent tumor cell intravasation in NSCLC. Furthermore, decreased TIMP-2 expression was shown to increase myeloid-derived suppressor cells in an A549 cell xenograft mouse model^37^. Therefore, high expression of *miR-130b* may affect the overall survival of patients with NSCLC by targeting *TIMP-2*, which in turn promotes cancer cell invasion and enhances the functions of endothelial cells and myeloid-derived suppressor cells in tumor tissues.

Our Ago2-IP analysis revealed over 300 candidate target genes of *miR-130b*, including phosphatase and tensin homolog deleted on chromosome 10 and FRZB (Supplementary Table 1). These genes have potential *miR-130b* binding sites within their 3′-UTRs. In our previous work, we reported that *miR-130b* promotes cell migration by targeting phosphatase and tensin homolog deleted on chromosome 10 in bladder cancer cells^22^. Frizzled-related protein, which functions as an antagonist of the Wnt pathway, exerts inhibitory effects on the epithelial-mesenchymal transition or migration activity in several cancers, including lung cancer^38,39^. Because *miR-130b* slightly promoted cell migration in A549 cells (Supplementary Fig. 3C), *miR-130b* may affect migration activity by targeting these genes in NSCLC cells. Recently, Tian *et al*. reported that *miR-130b* inhibition induces apoptosis in NSCLC cells via a peroxisome proliferator-activated receptor-γ/vascular endothelial growth factor A/BCL-2-mediated pathway^40^. Therefore, *miR-130b* may enhance the aggressiveness of lung cancer cells via targeting TIMP-2 or some other molecules in NSCLC cells.

SP1, which is reported as a transcriptional activator of *TIMP-2* gene in breast cancer, was also found as a candidate target gene of *miR-130b* (Supplementary Table 1)^41,42^. Although miR-130b mimic nor inhibitor had no significant effect on TIMP2 expression at the mRNA level in NSCLC cells, miR-130b might affect TIMP-2 expression at the transcriptional level via targeting Sp1 transcriptional factor in other types of cancer such as breast cancer.

In summary, we demonstrated that TIMP-2 was an important target molecule of *miR-130b in vitro* and in clinical tumor tissues and serum samples from patients with NSCLC. High expression of *miR-130b* downregulated TIMP-2 expression and enhanced MMP-2 activity to promote the invasion of NSCLC cells. These findings indicated that *miR-130b* functioned as an oncogenic miRNA in NSCLC and could therefore facilitate the development of molecular-targeted therapeutic drugs for NSCLC.

## Materials and Methods

### Analysis of TCGA miRNA expression data

Clinical information and RNA-sequencing data for the *miR-130* family in patients with adenocarcinoma (n = 122) and squamous cell carcinoma (n = 113) were obtained from TCGA database. Survival analysis was conducted by dividing patients into high (copy number: 1) and low (copy number: −1) groups based on *miR-130b*, *miR-301a*, and *miR-301b* expression. Overall survival rates for these two groups were evaluated by log-rank tests. The clinical information is presented in Table 2 (adenocarcinoma) and Supplementary Table 3 (squamous cell carcinoma).

**Table 2:** NSCLC clinical samples used in Fig. 1A–C.

Age (y)		Clinical stage	
median	66	I	36
range	41–86	II	68
**Gender**		III	10
male	57	IV	8
female	65		

### Clinical specimens

Specimens of NSCLC tissues and adjacent noncancerous tissues were obtained from patients who had undergone primary curative resection of lung tumors at Kagoshima University Hospital (Japan). All enrolled patients were diagnosed by pathological examination and staged by specialised oncologists via the 7th edition of the International Association for the Study of Lung Cancer TNM Classification. Prior written informed consent was obtained from all patients at Kagoshima University Hospital, and the methods were carried out in accordance with approved guidelines. Experiments using clinical specimens were approved by the institutional review boards of Kagoshima University Hospital and the Graduate School of Pharmaceutical Sciences, Osaka University. Clinical and histopathological data related to the clinical specimens are presented in Tables 1, 2 and 3.

**Table 3:** NSCLC clinical samples used in Fig. 5A–F.

Age (y)		Clinical stage	
median	72.5	I	34
range	35–88	II/III	19
**Gender**		unknown	1
male	36	**Operation**	
female	18	preoperation	24
**Vessel cancer cell invasion**		postoperation	24
v (−)	28	**Lymphatic cancer cell (ly) invasion**	
v (+)	25	ly (−)	28
unknown	1	ly (+)	25
**Pleura cancer cell invasion**		unknown	1
pl (−)	36		
pl (+)	18		

### RNA extraction

Clinical tissue samples were immediately immersed in RNAlater (Qiagen, Valencia, CA, USA) after operation and stored at −80 °C until RNA extraction. miRNAs were purified from NSCLC or adjacent normal tissue samples using an miRNeasy mini kit (Qiagen). For cell line experiments, total RNA was isolated using TRIzol reagent (Thermo Fisher Scientific, MA, USA) according to the manufacturer’s instructions.

### Real-time qPCR

cDNA was synthesised from total RNA (500 ng) in a 10-μl reaction volume using a PrimeScript RT-PCR Kit (TaKaRa, USA). Thermal cycling conditions included an initial step at 98 °C for 30 s, followed by 40 cycles at 95 °C for 5 s and 60 °C for 20 s. The following primers were used: *MMP-2* forward, 5′-GATAACCTGGATGCCGTCGTG-3′; *MMP-2* reverse, 5′-CAGCCTAGCCAGTCGGATTTG-3′; *TIMP-2* forward, 5′-TACCAGATGGGCTGCGAGTG-3′; *TIMP-2* reverse, 5′-CTGTGACCCAGTCCATCCAGAG-3′; and *GAPDH* forward, 5′-CCATCACCATCTTCCAGGAG-3′; *GAPDH* reverse, 5′-CCTGCTTCACCACCTTCTTG-3′.

The relative expression levels of mRNA were analysed using the 2^−ΔΔCt^ method. For miRNA analysis, cDNA was synthesised using a miR-X miRNA first-strand synthesis kit (TaKaRa, Japan). Thermal cycling conditions included an initial step at 98 °C for 30 s, followed by 40 cycles at 95 °C for 2 s and 63 °C for 5 s. The following primers were used: *miR-130b* primer, 5′-CAGTGCAATGATGAAAGGGCAT-3′; mRQ 3′ primer (TaKaRa); and U6 snRNA-specific primer (TaKaRa) as an internal control.

### Cell culture

A549 human lung cancer cells were maintained in Dulbecco’s modified Eagle’s medium (Wako, Japan) with 10% foetal bovine serum (FBS) and 100 mg/mL kanamycin. NCI-H520, NCI-H1755, NCI-H1975, 5637 and primary NSCLC cell lines were cultured in RPMI 1640 (Wako) with 10% FBS and 100 mg/mL kanamycin. HPL1D human lung epithelial cells were maintained in Ham’s F12 medium containing 10% FBS and 100 mg/mL kanamycin. Cells were cultured in an incubator at 37 °C in an atmosphere containing 5% CO_2_. A549 cells were purchased from Riken Cell Bank (Japan); NCI-H520, NCI-H1755, and NCI-H1975 cells were purchased from American Type Culture Collection (Manassas, VA, USA); and HPL1D and 5637 cells were kindly provided by the Department of Molecular Carcinogenesis, Nagoya University (Japan) and the Department of Urology, Osaka University (Japan), respectively.

### Plasmid construction

To construct TIMP-2 reporter plasmids, the following oligonucleotides were used:

human *TIMP-2* 3′-UTR oligo sense, 5′-CTAGCGGCCGCTAGTCCCTTGGTAGGTATTAGACTTGCACTTG-3′; human *TIMP-2* 3′-UTR oligo antisense, 5′-TCGACAAGTGCAAGTCTAATACCTACCAAGGGACTAGCGGCCGCTAGAGCT-3′; mutated *TIMP-2* 3′-UTR oligo sense, 5′-CTAGCGGCCGCTAGTCCCAACGTTGGATATTGACAACGTCACG-3′; and mutated *TIMP-2* 3′-UTR oligo antisense, 5′-TCGACGTGACGTTGTCAATATCCAACGTTGGGACTAGCGGCCGCTAGAGCT-3′. The annealed products were digested with *Sac*I and *Sal*I and inserted into a pmirGLO dual-luciferase miRNA target expression vector (Promega, Madison, WI, USA). The primary *hsa-miR-130b* and the coding domain sequence of *TIMP-2* were cloned from genomic DNA of 5637 cells and HPL1D cells, respectively, by PCR using KOD-FX (TOYOBO, Japan) and the following oligonucleotide primers: pri-*hsa-miR-130b* forward, 5′-TCGAAAGCTTTACCCAATTCGCTCCCTTCT-3′; pri-*hsa-miR-130b* reverse, 5′-TCGAGGATCCCACCCACCTGATCCTCTGAT-3′; *hsa-TIMP-2* forward, 5′-CGGAAGCTTATGGGCGCCGCGGCC-3′; and *hsa-TIMP-2* reverse, 5′-GGAGGCTCGAGTTATGGGTCCTCGATGTCG-3′. The PCR products were digested with *Hind*III and *BamH*I (*hsa-miR-130b*) or *Hind*III and *Xho*I (*TIMP-2*) and inserted into the pmR-ZsGreen1 miRNA expression vector (pmR-ZsGreen1/hsa-miR-130b) or pcDNA3.0 expression vector (pcDNA3.0/TIMP-2), respectively.

### Establishment of A549 cell clones with stable *miR-130b* overexpression

The pmR-ZsGreen1/pri-hsa-miR-130b vector was transfected into A549 cells seeded at 3 × 10^4^ cells/well in a 12-well plate using Lipofectamine 3000 (Invitrogen). At 24 h after transfection, culture medium was changed to new medium containing 1 mg/ml geneticin (Wako) to select transfected cells. The selected cells were subjected to a limited dilution method in 96-well plates (0.7 cells/well) to obtain a single colony. ZsGreen1-positive colonies were subjected to miRNA purification and then real-time qPCR to confirm the expression of *miR-130b*.

### Cell invasion assay

A tumor invasion assay system with an 8.0-μm pore size FluoroBlok membrane (Corning, NY, USA) was used to analyse cell invasion in A549 and NCI-H1755 cells. The cells were reseeded into inserts in 96-well plates (1.25 × 10^4^ cells/well) in serum-free conditions 24 h after transfection, and DMEM or RPMI1640 medium containing 10% FBS was added as a chemo-attractant in the base plate. A549 and NCI-H1755 cells were incubated for 20 and 48 h, respectively, at 37 °C in an atmosphere containing 5% CO_2_. After incubation, the cells were labelled with 4 μg/mL calcein AM (Dojindo, Japan), and the fluorescence intensity of the invaded cells was measured at wavelengths of 494/517 nm (excitation/emission) using an EnVision Multilabel Reader (PerkinElmer, USA).

### 3D invasion assay using primary NSCLC cells

We prepared neural stem/progenitor cells (NSC) medium containing DMEM/Ham’s F-12 (Wako), B-27^TM^ Supplement (Thermo Fisher), fibroblast growth factor human recombinant animal-derived free (Wako), heparin sulfate sodium salt from bovine kidney (Sigma Aldrich) and antibiotic-antimycotic (Thermo Fisher). Primary cultured NSCLC cells were seeded into Nunclon Sphera 96U-well plates (2000 cells/well) and cultured for 48 h. Half of the medium was replaced with NSC medium containing Matrigel and then incubated for 72 h at 37 °C in an atmosphere containing 5% CO_2_. Images were acquired at 72 h after the medium change using an OLYMPUS IX71 fluorescence microscope (Tokyo, Japan).

### Gelatine zymography

A549 cells were incubated at 37 °C in an atmosphere containing 5% CO_2_ in Dulbecco’s modified Eagle’s medium without foetal calf serum or antibiotics for 48 h. After incubation, conditioned medium was collected and concentrated using Amicon Ultra filters (Millipore, USA). Samples were mixed with Laemmli sodium dodecyl sulfate sample buffer without 2-mercaptoethanol and separated on 10% gelatine-containing gels. The gels were incubated in zymogram renaturing buffer (Invitrogen) at room temperature for 30 min and then in zymogram development buffer (Invitrogen) at 37 °C overnight. Gels were then washing and staining with Coomassie blue. Densitometric analysis was performed using NIH ImageJ software.

### Western blot analysis

Whole-cell lysates were separated by sodium dodecyl sulfate-polyacrylamide gel electrophoresis and then transferred to polyvinylidene difluoride membranes (Millipore) using a semidry transfer system (Bio-Rad, Hercules, CA, USA). The membranes were probed with specific antibodies and then incubated with horseradish peroxidase-conjugated antibodies against mouse or rabbit immunoglobulin (Santa Cruz Biotechnology, Santa Cruz, CA, USA), followed by detection with enhanced chemiluminescence western blotting detection reagent (GE Healthcare, IL USA). An ImageQuant LAS4000 mini system (GE Healthcare) was used as a chemiluminescence detector. The following antibodies were used in this study: anti-TIMP-2 (1:1000; cat. no. SAB1400279; Sigma-Aldrich, St. Louis, MO, USA), anti-MMP-2 (1:2000; cat. no. 13132; Cell Signaling Technology, Danvers, MA, USA), and anti-β-actin (polyclonal; 1:50000; cat. no. A5316; Sigma-Aldrich). Densitometric analysis was performed using NIH Image J software.

### Dual-luciferase assay

A pmirGLO dual-luciferase miRNA target expression vector was used for luciferase reporter assays (Promega). A549 cells were transfected with the reporter vector containing the predicted *miR-130b* binding site or mutated *miR-130b* binding site in the *TIMP-2* 3′-UTR (Supplementary Fig. 4B). After transfection for 24 h, dual-luciferase reporter assays were performed using a luminometer (Turner Biosystems 20/20 luminometer; Promega) according to the manufacturer’s protocol.

### Transfection

MiRIDIAN miRNA mimic for *hsa-miR-130b-3p* (C-300660-05-000), miRNA mimic negative control (CN-001000-01-05), miRIDIAN miRNA hairpin inhibitor for *hsa-miR-130b-3p* (IH-300660-07-0005), and miRNA hairpin inhibitor negative control (IN-001005-01-05) were purchased from GE Healthcare. The miRNA mimic and the hairpin inhibitor were transfected at a concentration of 50 nM using Lipofectamine 3000 (Invitrogen). These transfection experiments were performed according to the protocol supplied by the manufacturer.

### ELISA

A human TIMP-2 ELISA kit (Sigma-Aldrich) was used to detect TIMP-2 levels in the serum of patients with NSCLC according to the manufacturer’s protocol. Serum from patients with NSCLC was diluted 2000-fold before use, and colour intensity was measured at 450 nm using an Envision Multilabel Reader (PerkinElmer, MA, USA). Clinical information of specimens used for ELISA is presented in Table 3.

### Statistics

F-test was preliminarily performed for verifying equality of variance. For all the other experiments, results were expressed as the mean ± standard deviation of the mean (S.D.) or median. Differences between values were statistically analysed using Student’s t-tests or one-way analysis of variance with Bonferroni post-hoc tests as appropriate (GraphPad Prism 5.0; GraphPad, USA). Differences with *p* values of less than 0.05 were considered statistically significant.

## Supplementary information

Supplementary information
