# Supplementary material for: MicroRNA-130b functions as an oncomiRNA in non-small cell lung cancer by targeting tissue inhibitor of metalloproteinase-2
Source: Sci Rep. 2019 May 6;9:6956. doi: 10.1038/s41598-019-43355-8 (PMC6502853; doi:10.1038/s41598-019-43355-8)
Supplement: Supplementary file 1 — Supplementary information [file 41598_2019_43355_MOESM1_ESM.pdf]

MicroRNA-130b functions as an oncomiRNA in non-small cell lung cancer by targeting tissue inhibitor of metalloproteinase-2

Takayuki Hirono, Kentaro Jingushi, Toshiyuki Nagata, Masami Sato, Kentaro Minami, Masaya Aoki, Aya Harada Takeda, Tadashi Umehara, Hiroshi Egawa, Yoshino Nakatsuji, Kaori Kitae, Yuko Ueda, Hiroaki Hase, Masatatsu Yamamoto, Yoshinari Shinsato, Kohichi Kawahara, Tatsuhiko Furukawa, Kazutake Tsujikawa

Supplementary Material and Methods

Cell culture

HPL1D, HLC-1, and EBC-1 cells were maintained in Ham's F12 medium (Wako, Japan). PC-9, NCI-H23, NCI-H2228, NCI-H1437, II-18, NCI-H1792, RERF-LC-KJ, LC-2/ad, NCI-H1838, NCI-H226, and Sq-1 cells were cultured in RPMI 1640 medium (Wako). Calu-3 cells and RERF-LC-AI cells were maintained in Eagle's minimum essential medium (Wako) and minimum essential medium, respectively. The medium was supplemented with 10% FBS and 100 mg/mL kanamycin. The cells were incubated at 37°C in an atmosphere containing 5% CO_2. HLC-1, EBC-1, Sq-1, LC-2/ad, RERF-LC-AI, and II-18 cells were purchased from Riken Cell Bank (Japan). PC-9 cells were purchased from Cell Resource Center for Biomedical Research and Cell Bank, Tohoku University (Japan). NCI-H23, NCI-H2228, NCI-H1650, NCI-H1437, NCI-H1792, RERF-LC-KJ, Calu-3, NCI-H1838, and NCI-H1226 cells were purchased from American Type Culture Collection (USA).

Water-soluble tetrazolium salt-8 (WST-8) cell proliferation assays

Cell proliferation was examined by WST-8 assays. NCI-H1755 cells transfected with miR-130b inhibitor were reseeded in 96-well plates (3000 cells/well) for anchorage-dependent assays. A549 cells stably overexpressing *miR-130b* were seeded

in 96-well plates (500 cells/well) for anchorage-dependent assays or Nunclon Sphera 96F-well plates (1000 cells/well; Nunc) for anchorage-independent assays. Cells were then incubated for the indicated times, and WST-8 reagent (Dojindo, Japan) was then added. Cells were incubated for 2 h at 37°C in an atmosphere containing 5% CO_2, and the optical density was determined at a wavelength of 450/630 nm (measurement/reference) using an iMark microplate reader (Bio-Rad, Hercules, CA, USA).

Wound healing assay

A549 cells stably overexpressing *miR-130b* were seeded in 24-well plates (1×10^5 cells/well) and incubated for 48 h. A wound was created in the cell monolayer at ~90% confluency using a sterile 1-ml pipette tip. Images were acquired at 0 and 48 h after wound creation using an OLYMPUS IX71 fluorescence microscope (Olympus, Japan).

RNA extraction

miRNAs were purified from NSCLC cell line 24h after transfection using an miRNeasy mini kit (Qiagen). Total RNA was isolated 24h after transfection using TRIzol reagent (Thermo Fisher Scientific, MA, USA) according to the manufacturer's instructions.

Cell invasion assay

NCI-H520 cells were reseeded into inserts in 96-well plates (1.25×10^4 cells/well) in

serum-free conditions 24 h after transfection. NCI-H520 cells were incubated for 24 at 37°C in an atmosphere containing 5% CO_2.

Ago2-IP and gene array analysis

Ago2-IP was performed using an IP kit (RNA-binding protein IP-assay kit for microRNA; MBL, Japan) following the manufacturer's instructions. Briefly, RNAs immunoprecipitated with anti-ago2 antibodies were eluted from beads with hemagglutinin peptide (Wako), and QIAzol reagent (Qiagen) was added to extract RNAs. Ago2-bound RNAs were cleaned using a miRNeasy column and then subjected to microarray analysis. After confirming the purity of total RNAs by Experion (Bio-Rad), cDNA was synthesized using a WT expression kit (Applied Biosystems, Foster City, CA, USA), biotinylated using a GeneChip WT terminal labeling kit (Affymetrix, USA), and then injected into a GeneChip Human 1.0 ST array (Affymetrix). After hybridization, the GeneChip was washed and stained using a GeneChip hybridization wash and stain kit (Affymetrix) with a GeneChip fluidics station (Affymetrix). The stained GeneChip was then scanned using a GeneChip scanner 3000 (Affymetrix). Partek Genomics Suite 6.6 (Molsis, Japan) was used for data analysis (GEO accession number: GSE118274).

Western blot analysis

A549, NCI-H520 and NCI-H1975 cells were transfected with the negative control mimic or *miR-130b* mimic for 48 h, and whole-cell lysates were used for western blot analysis. The following antibodies were used in this study: anti-TIMP-2 (1:1000; cat. no. SAB1400279; Sigma-Aldrich, St. Louis, MO, USA), and anti-β-actin (polyclonal; 1:50000; cat. no. A5316; Sigma-Aldrich). Densitometric analysis was performed using NIH Image J software.

Supplementary Table 1

Sample	mock	130b
Cell number (cells)	167.5±0.5×10^4	161.5±3.5×10^4
Protein amount in cultured-medium (μg)	453.3±9.1	437.1±6.7

Supplementary Table 2

List of genes identified by Ago2-IP and gene array analysis

Gene symbol	Gene name
ACSL4	acyl-CoA synthetase long-chain family member 4
ACVR1C	activin A receptor, type IC
ADAM12	ADAM metallopeptidase domain 12
ALX4	ALX homeobox 4
ANKFY1	ankyrin repeat and FYVE domain containing 1
ANKRD13C	ankyrin repeat domain 13C
ANKRD52	ankyrin repeat domain 52
APCDD1	adenomatosis polyposis coli down-regulated 1
AQPEP	laeverin
ARHGAP24	Rho GTPase activating protein 24
ARID4B	AT rich interactive domain 4B (RBP1-like)
ARL4A	ADP-ribosylation factor-like 4A
ARL6IP1	ADP-ribosylation factor-like 6 interacting protein 1
ARRDC3	arrestin domain containing 3
ATP11A	ATPase, class VI, type 11A
ATP13A3	ATPase type 13A3
ATP2B2	ATPase, Ca++ transporting, plasma membrane 2
B4GALT5	UDP-Gal:betaGlcNAc beta 1,4- galactosyltransferase, polypeptide5
BACH2	BTB and CNC homology 1, basic leucine zipper transcription factor 2
BAG5	BCL2-associated athanogene 5
BAHD1	bromo adjacent homology domain containing 1
BARHL2	BarH-like homeobox 2
BAZ1A	bromodomain adjacent to zinc finger domain, 1A
BCL2L11	BCL2-like 11 (apoptosis facilitator)

BHLHE41	basic helix-loop-helix family, member e41
BMPR2	bone morphogenetic protein receptor, type II
BPTF	bromodomain PHD finger transcription factor
BRWD1	bromodomain and WD repeat domain containing 1
BZRAP1	benzodiazapine receptor (peripheral) associated protein 1
C5orf30	chromosome 5 open reading frame 30
C7orf60	chromosome 7 open reading frame 60
CAST	calpastatin
CBX6	chromobox homolog 6
CCDC88A	coiled-coil domain containing 88A
CCNA2	cyclin A2
CCND3	cyclin D3
CD69	CD69 molecule
CDK19	cyclin-dependent kinase 19
CENPO	centromere protein O
CEP55	centrosomal protein 55kDa
CFL2	cofilin 2
CHD5	chromodomain helicase DNA binding protein 5
CHRM2	cholinergic receptor, muscarinic 2
CHST1	carbohydrate (keratan sulfate Gal-6) sulfotransferase 1
CMPK2	cytidine monophosphate (UMP-CMP) kinase 2, mitochondrial
CNOT6	CCR4-NOT transcription complex, subunit 6
CNOT6L	CCR4-NOT transcription complex, subunit 6-like
COL19A1	collagen, type XIX, alpha 1
COL9A3	collagen, type IX, alpha 3
COX8C	cytochrome c oxidase subunit 8C
CPEB2	cytoplasmic polyadenylation element binding protein 2
CREB5	cAMP responsive element binding protein 5
CSMD1	CUB and Sushi multiple domains 1
CUL3	cullin 3
CYP2U1	cytochrome P450, family 2, subfamily U, polypeptide 1
DCUN1D3	DCN1, defective in cullin neddylation 1, domain containing 3
DDX6	DEAD (Asp-Glu-Ala-Asp) box polypeptide 6
DEPDC1	DEP domain containing 1
DGCR14	DiGeorge syndrome critical region gene 14
DICER1	DICER1 ribonuclease Ⅲ

DLC1	deleted in liver cancer 1
DSEL	dermatan sulfate epimerase-like
DYNLL2	dynein, light chain, LC8-type 2
DYRK2	dual-specificity tyrosine-(Y)-phosphorylation regulated kinase 2
E2F2	E2F transcription factor 2
E2F7	E2F transcription factor 7
EFNA5	ephrin-A5
EFNB2	ephrin-B2
EGR3	early growth response 3
EIF4E3	eukaryotic translation initiation factor 4E family member 3
ENPP5	ectonucleotide pyrophosphatase/phosphodiesterase 5
ENPP6	ectonucleotide pyrophosphatase
EPB41L1	erythrocyte membrane protein band 4.1-like 1
ESR1	estrogen receptor 1
ESR1	estrogen receptor 1
ETV3	ets variant 3
EXOC5	exocyst complex component 5
EXOC5	exocyst complex component 5
EXOC5	exocyst complex component 5
FAM107B	family with sequence similarity 107, member B
FAM13A	family with sequence similarity 13, member A
FAM43A	Nfamily with sequence similarity 43, member A /
FANCA	Fanconi anemia, complementation group A
FGF10	fibroblast growth factor 10
FMNL3	formin-like 3
FMR1	fragile X mental retardation 1
FOSL2	FOS-like antigen 2
FRZB	frizzled-related protein
FUBP1	far upstream element (FUSE) binding protein 1
FXR1	fragile X mental retardation, autosomal homolog 1
G3BP2	GTPase activating protein (SH3 domain) binding protein 2
GAN	gigaxonin
GATAD2B	GATA zinc finger domain containing 2B
GBX2	gastrulation brain homeobox 2
GGA2	golgi-associated, gamma adaptin ear containing, ARF binding protein 2
GLRA3	glycine receptor, alpha 3

GMEB1	glucocorticoid modulatory element binding protein 1
GMFB	glia maturation factor, beta
GPC6	glypican 6
GPR158	G protein-coupled receptor 158
HADHA	Hydroxyacyl-Coenzyme A Dehydrogenase/3-Ketoacyl-Coenzyme A Thiolase/Enoyl-Coenzyme A Hydratase, Alpha Subunit
HOXA5	homeobox A5
HOXB1	homeobox B1
HOXB2	homeobox B2
HOXD1	homeobox D1
HSPA8	heat shock 70kDa protein 8
IMPDH1	IMP (inosine 5'-monophosphate) dehydrogenase 1
INSIG1	insulin induced gene 1
IRF1	interferon regulatory factor 1
ITGA11	integrin, alpha 11
ITGA4	integrin, alpha 4
ITPK1	inositol 1,3,4-triphosphate 5/6 kinase
ITPKB	inositol 1,4,5-trisphosphate 3-kinase B
JARID2	jumonji, AT rich interactive domain 2
KALRN	kalirin, RhoGEF kinase
KCNA4	potassium voltage-gated channel, shaker-related subfamily A, member 4
KCNJ15	potassium inwardly-rectifying channel, subfamily J, member 15
KCNJ6	potassium inwardly-rectifying channel, subfamily J, member 6
KCNK10	potassium channel, subfamily K, member 10
KCNN3	potassium intermediate/small conductance calcium-activated channel subfamily N member 3
KDM2A	lysine (K)-specific demethylase 2A
KIAA1217	KIAA1217
KIF13A	kinesin family member 13A
KLF6	Kruppel-like factor 6
KLHDC8A	kelch domain containing 8A
LBR	lamin B receptor
LBR	lamin B receptor
LDLR	low density lipoprotein receptor
LIMD2	LIM domain containing 2
LRCH2	leucine-rich repeats and calponin homology (CH) domain containing 2

LRP2	ow density lipoprotein receptor-related protein 2
LRP4	low density lipoprotein receptor-related protein 4
LRP6	low density lipoprotein receptor-related protein 6
LRP8	low density lipoprotein receptor-related protein 8, apolipoprotein E receptor
LZIC	leucine zipper and CTNNBIP1 domain containing
MAP4K4	mitogen-activated protein kinase kinase kinase kinase 4
MAP7	microtubule-associated protein 7
MAPK1	mitogen-activated protein kinase 1
MDGA2	MAM domain containing glycosylphosphatidylinositol anchor 2
MECP2	methyl CpG binding protein 2 (Rett syndrome)
MEMO1	mediator of cell motility 1
MEMO1	mediator of cell motility 1
MEOX2	mesenchyme homeobox 2
MIER3	mesoderm induction early response 1, family member 3
MPPED2	metallophosphoesterase domain containing 2
MTMR12	myotubularin related protein 12
MYBL1	v-myb myeloblastosis viral oncogene homolog (avian)-like 1
MYO10	myosin X
MYT1L	myelin transcription factor 1-like
NAA30	N(alpha)-acetyltransferase 30, NatC catalytic subunit
NAA50	N(alpha)-acetyltransferase 50, NatE catalytic subunit
NACC2	NACC family member 2, BEN and BTB (POZ) domain containing
NAP1L3	nucleosome assembly protein 1-like 3
NCKAP5	NCK-associated protein 5
NCOA1	nuclear receptor coactivator 1
NDRG2	NDRG family member 2
NEUROD1	neurogenic differentiation 1
NEUROG1	neurogenin 1
NFATC2	nuclear factor of activated T-cells, cytoplasmic, calcineurin-dependent 2
NFIA	nuclear factor I/A
NFIC	nuclear factor I/C (CCAAT-binding transcription factor)
NHLH2	nescient helix loop helix 2
NHSL2	NHS-like 2
NPAT	nuclear protein, ataxia-telangiectasia locus
NPNT	nephronectin
NPTX1	neuronal pentraxin I

NRARP	NOTCH-regulated ankyrin repeat protein
NRP1	neuropilin 1
NUP133	nucleoporin 133kDa
OSTF1	osteoclast stimulating factor 1
OTUD3	OTU domain containing 3
PCYT1B	phosphate cytidylyltransferase 1, choline, beta
PCYT2	phosphate cytidylyltransferase 2, ethanolamine
PCYT2	phosphate cytidylyltransferase 2, ethanolamine
PDE4D	NM_001165899 // PDE4D // phosphodiesterase 4D, cAMP-specific (phosphodiesterase E3 dunc
PDE5A	phosphodiesterase 5A, cGMP-specific
PEX5L	peroxisomal biogenesis factor 5-like
PFKFB3	6-phosphofructo-2-kinase/fructose-2,6-biphosphatase 3
PGM2L1	phosphoglucomutase 2-like 1
PHF14	PHD finger protein 14
PHF20	PHD finger protein 20
PITPNM2	phosphatidylinositol transfer protein, membrane-associated 2
PMEPA1	prostate transmembrane protein, androgen induced 1
POU3F2	POU class 3 homeobox 2
POU6F1	POU class 6 homeobox 1
PPP2R5E	protein phosphatase 2, regulatory subunit B', epsilon isoform
PRKACB	protein kinase, cAMP-dependent, catalytic, beta
PRKD3	protein kinase D3
PRKG1	protein kinase, cGMP-dependent, type I
PRR5L	proline rich 5 like
PSD	pleckstrin and Sec7 domain containing
PTBP2	polypyrimidine tract binding protein 2
PTEN	phosphatase and tensin homolog
PTGES3	prostaglandin E synthase 3 (cytosolic)
PTGFRN	prostaglandin F2 receptor negative regulator
PTP4A1	protein tyrosine phosphatase type IVA, member 1
PTPN14	protein tyrosine phosphatase, non-receptor type 14
PURG	purine-rich element binding protein G
PVRL3	poliovirus receptor-related 3
QSER1	glutamine and serine rich 1
RAB14	RAB14, member RAS oncogene family

RAB9B	RAB9B, member RAS oncogene family
RACGAP1	Rac GTPase activating protein 1
RALBP1	ralA binding protein 1
RAPGEF4	Rap guanine nucleotide exchange factor (GEF) 4
RASD1	RAS, dexamethasone-induced 1
RASSF2	Ras association (RalGDS/AF-6) domain family member 2
RBM17	RNA binding motif protein 17
RFX7	regulatory factor X, 7
RLIM	ring finger protein, LIM domain interacting
RND2	Rho family GTPase 2
RNF213	ring finger protein 213
RNF38	ring finger protein 38
ROCK2	Rho-associated, coiled-coil containing protein kinase 2
RPA2	replication protein A2, 32kDa
RPS6KA5	ribosomal protein S6 kinase, 90kDa, polypeptide 5
RTN1	reticulon 1
RUNDC1	RUN domain containing 1
RXRA	retinoid X receptor, alpha
S1PR2	sphingosine-1-phosphate receptor 2
SAMD8	sterile alpha motif domain containing 8
SERBP1	SERPINE1 mRNA binding protein 1
SESTD1	SEC14 and spectrin domains 1
SGCB	sarcoglycan, beta (43kDa dystrophin-associated glycoprotein)
SHC3	SHC (Src homology 2 domain containing) transforming protein 3
SIK1	salt-inducible kinase 1
SIX4	SIX homeobox 4
SKP1	S-phase kinase-associated protein 1
SLC13A2	solute carrier family 13A2
SLC24A3	solute carrier family 24 memberA3
SLC25A12	solute carrier family 25 member A12
SLC25A44	solute carrier family 25, member 44
SLC39A10	solute carrier family 39 member 10
SLC44A1	solute carrier family 44, member 1
SLC6A6	solute carrier family 6 member A6
SMAD5	SMAD family member 5
SMOC2	SPARC related modular calcium binding 2

SMURF2	SMAD specific E3 ubiquitin protein ligase 2
SNIP1	Smad nuclear interacting protein 1
SOCS6	suppressor of cytokine signaling 6
SOX21	SRY (sex determining region Y)-box 21
SOX5	SRY (sex determining region Y)-box 5
SP1	Sp1 transcription factor
SPOCK1	osteonectin, cwcv and kazal-like domains proteoglycan1
ST18	suppression of tumorigenicity 18
ST8SIA5	ST8 alpha-N-acetyl-neuraminide alpha-2,8-sialyltransferase 5
STAT3	signal transducer and activator of transcription 3
STC1	stanniocalcin 1
STX6	syntaxin 6
SULF1	sulfatase 1
SYNCRIP	synaptotagmin binding, cytoplasmic RNA interacting protein
SYNM	synemin, intermediate filament protein
SYT10	synaptotagmin X
TACC1	transforming, acidic coiled-coil containing protein 1
TAF4	TAF4 RNA polymerase II, TATA box binding protein (TBP)-associated factor
TBL1XR1	transducin (beta)-like 1 X-linked receptor 1
TCF4	transcription factor 4
TESK2	testis-specific kinase 2
TEX2	testis expressed 2
TEX261	testis expressed 261
TFDP2	transcription factor Dp-2 (E2F dimerization partner 2)
TGFB2	transforming growth factor, beta 2
TGFBR1	transforming growth factor, beta receptor 1
TGFBR2	transforming growth factor, beta receptor II (70/80kDa)
TGFBRAP1	transforming growth factor, beta receptor associated protein 1
TGOLN2	trans-golgi network protein 2
THRA	thyroid hormone receptor, alpha
THSD7A	thrombospondin, type I, domain containing 7A
TIMP2	TIMP metallopeptidase inhibitor 2
TMEM151B	transmembrane protein 151B
TNFRSF1B	tumor necrosis factor receptor superfamily, member 1B
TNFSF10	tumor necrosis factor (ligand) superfamily, member 10
TNRC6A	trinucleotide repeat containing 6A

TNRC6B	trinucleotide repeat containing 6B
TNRC6C	trinucleotide repeat containing 6C
TP63	tumor protein p63
TRERF1	transcriptional regulating factor 1
TRERF1	transcriptional regulating factor 1
TRIM3	tripartite motif-containing 3
TSC22D1	TSC22 domain family, member 1
TSHZ1	teashirt zinc finger homeobox 1
UBAP2L	ubiquitin associated protein 2-like
UCP3	uncoupling protein 3 (mitochondrial, proton carrier)
UHMK1	U2AF homology motif (UHM) kinase 1
USP28	ubiquitin specific peptidase 28
USP33	ubiquitin specific peptidase 33
USP46	ubiquitin specific peptidase 46
VAV2	vav 2 guanine nucleotide exchange factor
VCPIP1	valosin containing protein (p97)/p47 complex interacting protein
VSIG10	V-set and immunoglobulin domain containing 10
WDR20	WD repeat domain 20
WNK3	WNK lysine deficient protein kinase 3
WNT2B	wingless-type MMTV integration site family, member 2B
ZBTB20	zinc finger and BTB domain containing 20
ZBTB37	zinc finger and BTB domain containing 37
ZBTB7A	zinc finger and BTB domain containing 7A
ZCCHC14	zinc finger, CCHC domain containing 14
ZCCHC24	zinc finger, CCHC domain containing 24
ZFC3H1	zinc finger, C3H1-type containing
ZMAT3	zinc finger, matrin type 3
ZNF275	zinc finger protein 275
ZNF3	zinc finger protein 3
ZNF652	zinc finger protein 652
ZNF704	zinc finger protein 704
ZNF784	zinc finger protein 784
ZNRF3	zinc and ring finger 3

Supplementary Table 3

NSCLC clinical samples used in Supplementary Figs. 1A-C

**Age (y)**		**Clinical stage**	
median	68	I	24
range	39-90	II	71
**Gender**		III	10
male	84	IV	8
female	29		

Supplementary Figures

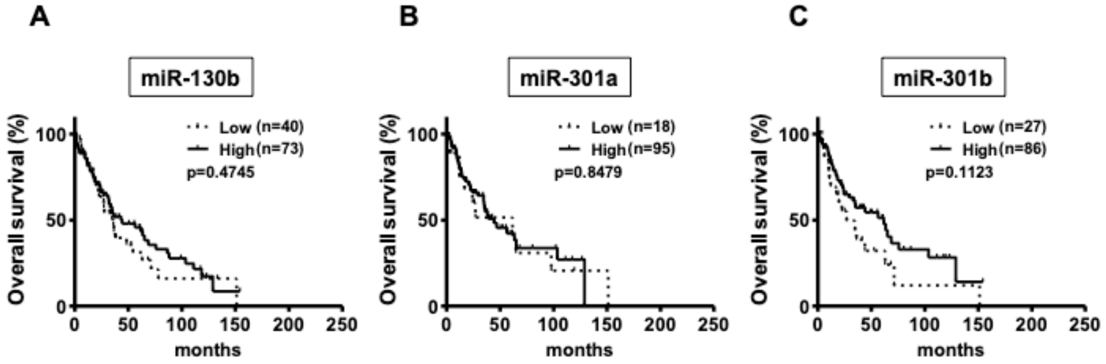

Supplementary Figure 1. Relationship between miR-130 family expression levels with overall survival of patients with squamous cell carcinoma.

(A–C) TCGA database analysis of *miR-130b*, *miR-301a*, and *miR-301b* in patients with squamous cell carcinoma. Overall survival analysis of patients with high (copy number: 1) and low (copy number: -1) *miR-130* family expression was analyzed by Kaplan-Meier analysis with log-rank tests. The number of patients analyzed is indicated in parentheses.

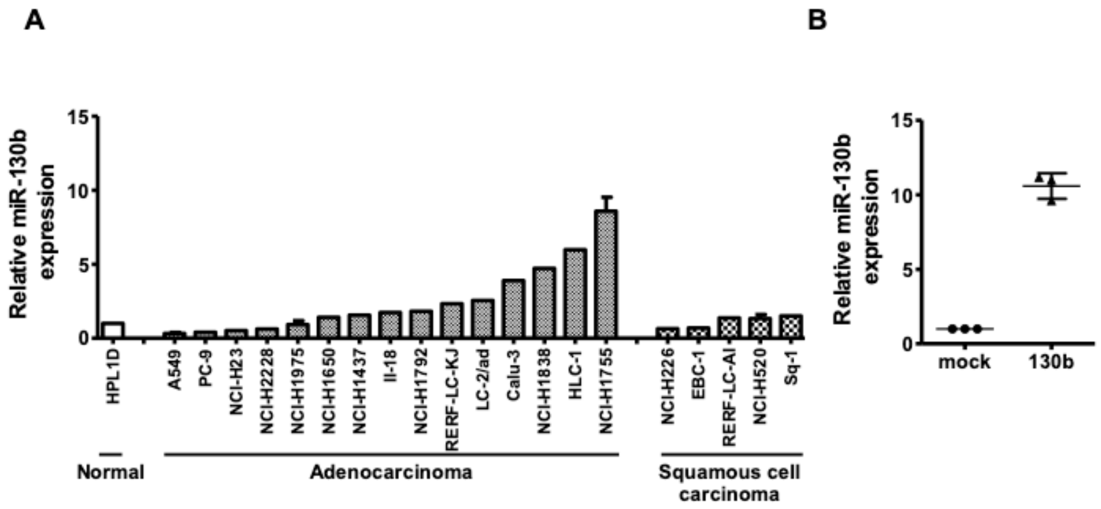

Supplementary Figure 2. *miR-130b* expression in NSCLC cell lines.

Expression of *miR-130b* in NSCLC cell lines (A) and in a clone isolated from A549 cells stably expressing *miR-130b* (B) was examined by real-time qPCR. Relative expression of *miR-130b* normalized to U6 snRNA is shown from duplicate experiments. In A549, NCI-H1975, NCI-H1755, and NCI-H520 cells, data show the means ± standard deviations of three independent experiments.

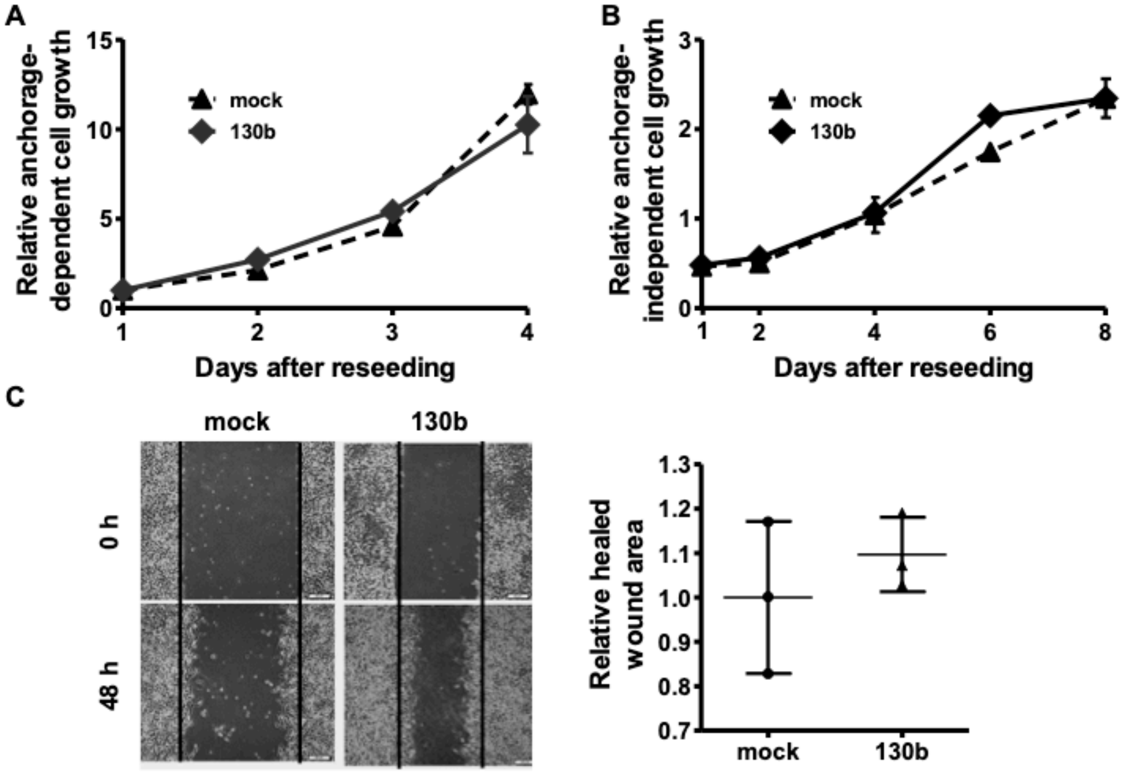

Supplementary Figure 3. Effects of *miR-130b* on the growth and migration of A549 cells.

(A, B) A549 cells stably overexpressing *miR-130b* were seeded in 96-well plates (A) or Nunclon Sphera 96F-well plates (B) and incubated for the indicated times. Relative cell proliferation was measured by WST-8 assays. Data are means ± standard deviations of three independent experiments. (C) Relative cell motility was measured 48 h using scratch wound assays. Representative results of cell motility are shown in the left panels. Data are means ± standard deviations of three independent experiments (right panel).

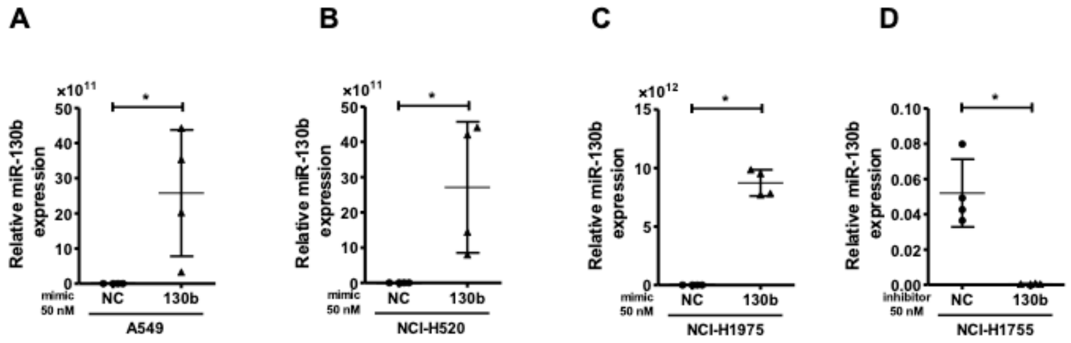

Supplementary Figure 4. *miR-130b* expression in miR-130b mimic or miR-130b inhibitor-transfected NSCLC cell lines.

miR-130b expression in miR-130b mimic or inhibitor-transfected NSCLC cell lines (A549, NCI-H520, NCI-H1975, and NCI-H1755 cells) were determined by real-time qPCR. Data are shown as the mean ± S.D. of three independent experiments.

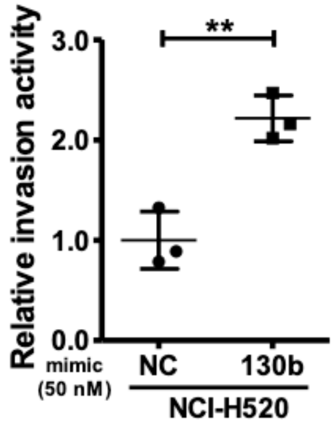

Supplementary Figure 5. Effect of *miR-130b* mimic on invasion activity in NCI-H520 cells.

NCI-H520 cells transfected with the *miR-130b* mimic (130b) or negative control miRNA mimic (NC) were subjected to invasion assays. The transfected cells were added to the upper chambers of Matrigel-coated transwell membrane inserts, and the lower chambers were filled with medium. Cells were cultured for 24 h. Fluorescence derived from invasive cells was measured. Data are the means ± standard deviations of three independent experiments. ***p* < 0.01 for t-tests.

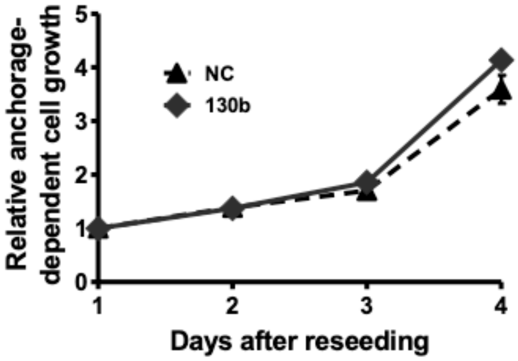

Supplementary Figure 6. Effects of *miR-130b* inhibitor on growth of NCI-H1755 cells.

NCI-H1755 cells transfected with the *miR-130b* inhibitor (130b) or negative control miRNA inhibitor (NC) were seeded into 96-well plates and incubated for the indicated times. Relative cell proliferation was measured by WST-8 assays. Data are the means ± standard deviations of three independent experiments.

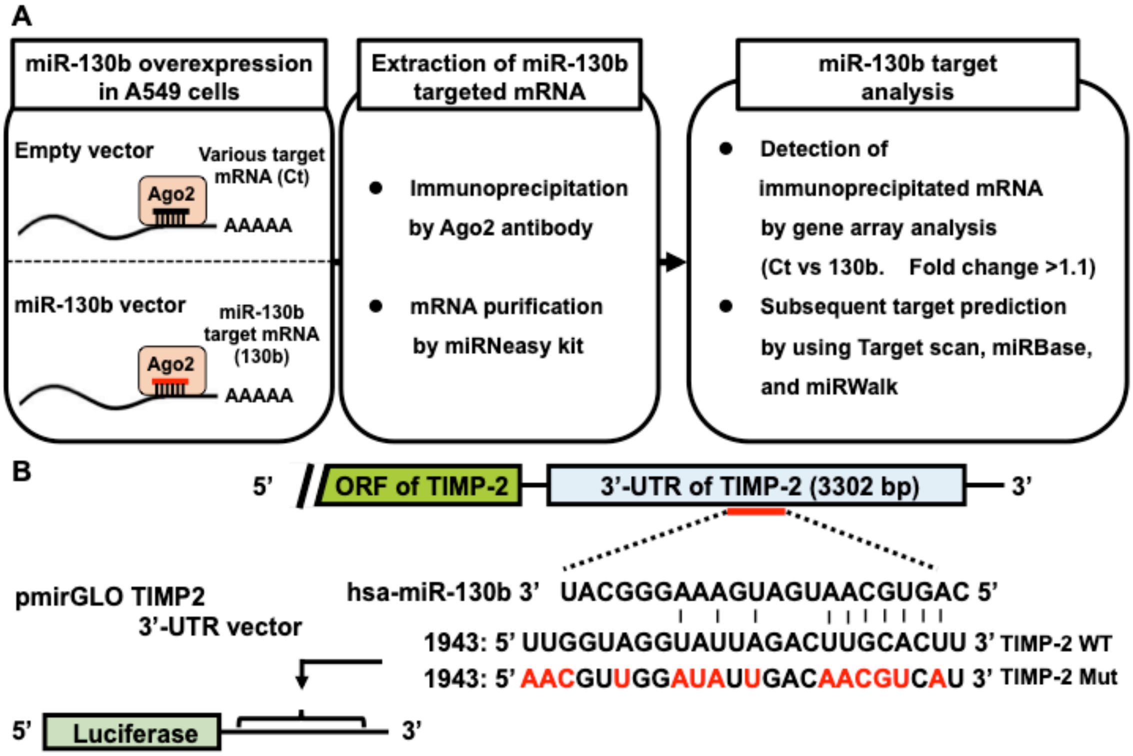

Supplementary Figure 7. Schematic illustration of the method used for identification of *miR-130b* target molecules by Ago2-IP.

miR-130b targets were analysed using a combination of Ago2-IP, gene array analysis, and database search. (B) Sequences of predicted *miR-130b* binding sites (wild-type TIMP-2) and the mutated *miR-130b*-binding site (mutant TIMP-2) in the human *TIMP-2* gene are shown. Nucleic acid bases mutated in the *TIMP-2* 3′-UTR are shown in red.

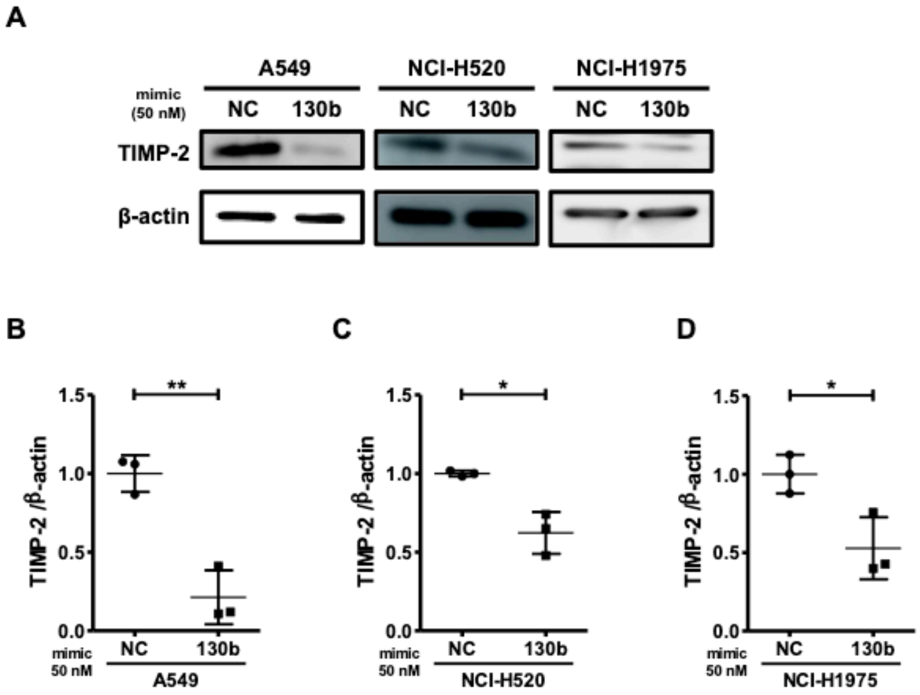

Supplementary Figure 8. Effect of miR-130b mimic on TIMP-2 expression in NSCLC cells.

(A) A549, NCI-H520 and NCI-H1975 cells were transfected with the negative control mimic (NC) or *miR-130b* mimic (130b) for 48 h, and whole-cell lysates were subjected to western blotting analysis with anti-TIMP-2 antibodies. (B-D) Densitometric analysis of the western blot results was performed using NIH ImageJ software and the results are shown as the TIMP-2 /β-actin ratio. *p < 0.05, **p < 0.01 for Student's t-test. Uncropped western blot data is shown in Supplementary Fig. 10.

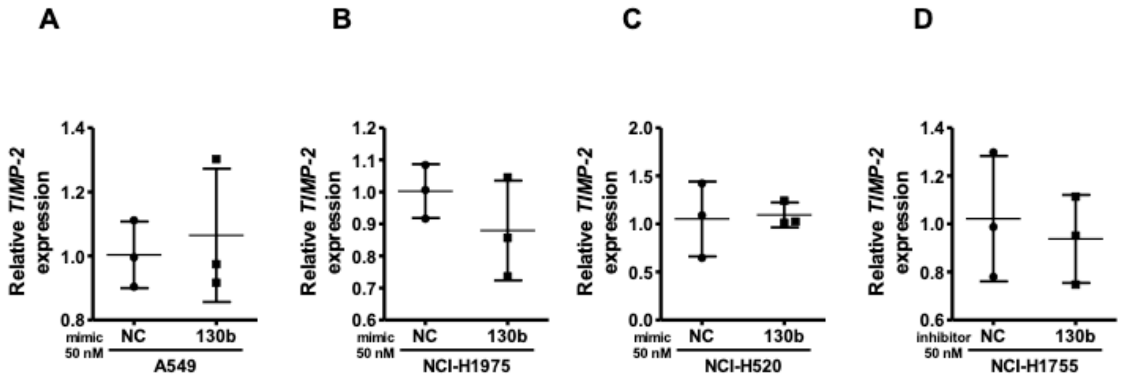

Supplementary Figure 9. Effect of miR-130b mimic or miR-130b inhibitor on *TIMP-2* mRNA expression in NSCLC cells.

(A-D) A549, NCI-H520 and NCI-H1975 cells transfected with the *miR-130b* mimic (130b) or negative control miRNA mimic (NC), or NCI-H1755 cells transfected with the *miR-130b* inhibitor (130b) or negative control miRNA inhibitor (NC) were subjected to real-time qPCR analysis of *TIMP-2*. Relative expression of *TIMP-2* normalized to *GAPDH* is shown as the means ± standard deviations of three independent experiments.

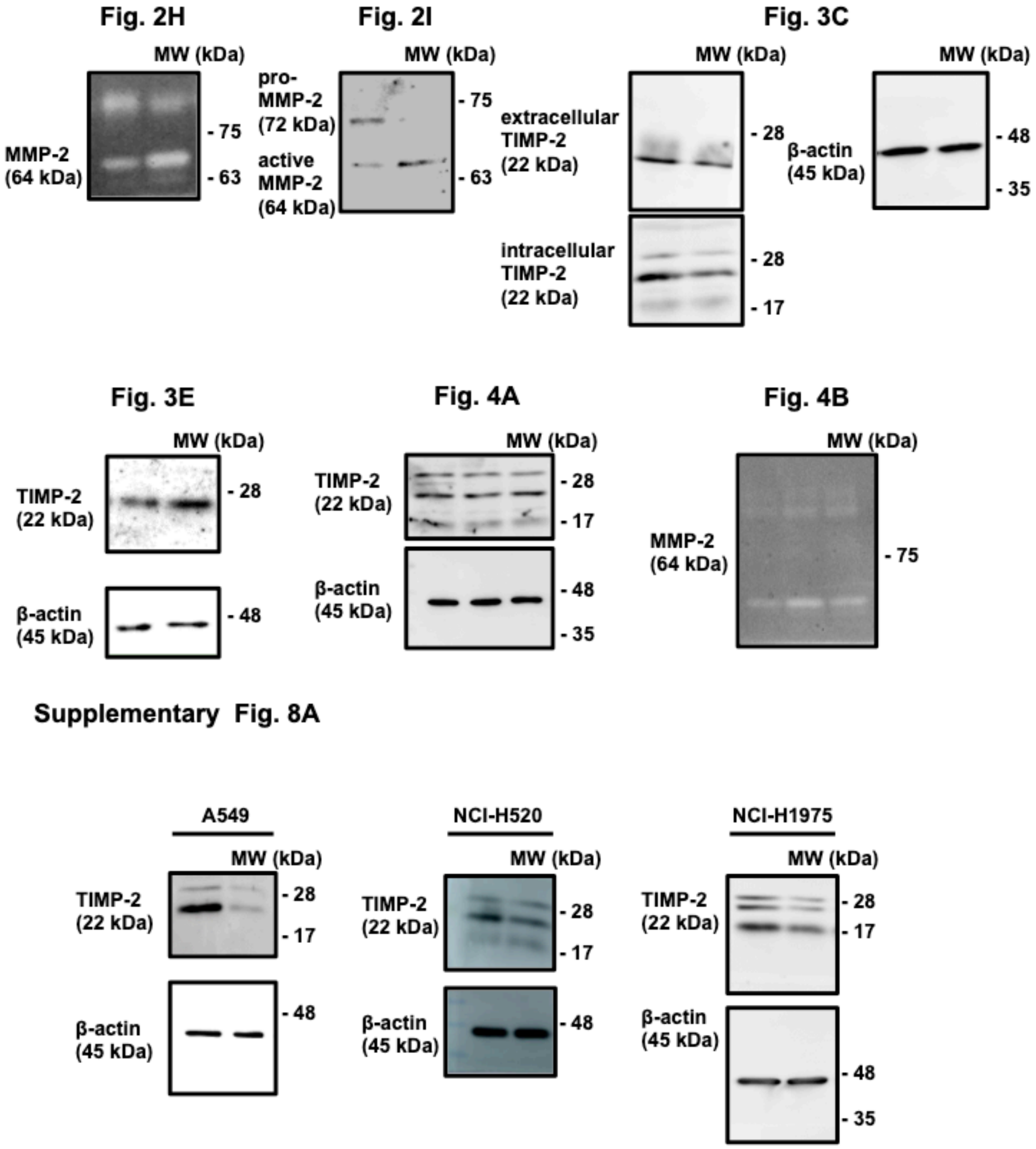

Supplementary Figure 10. Uncropped western blot data for Fig. 2H, 2I, 3C, 3E, 4A, 4B and Supplementary Fig. 8A.
